# Kinetic gait analysis in English Bulldogs

**DOI:** 10.1186/s13028-017-0344-6

**Published:** 2017-11-02

**Authors:** Andrés Sebastian Aristizabal Escobar, Alexandre Navarro Alves de Souza, Ana Carolina Brandão de Campos Fonseca Pinto, Julia Maria Matera

**Affiliations:** Department of Surgery, School of Veterinary Medicine and Animal Science, University of São Paulo (FMVZ/USP), São Paulo, SP Brazil

**Keywords:** Canine locomotion, English Bulldog, Kinetic, Orthopedic, Symmetry index

## Abstract

**Background:**

Canine breed conformation may interfere with locomotion and may predispose to orthopedic disease. Bulldogs have a high incidence of orthopedic diseases such as hip dysplasia. Kinetic gait analysis provides an objective way to assess and analyze locomotion. The aim of this study was to study the vertical forces of English Bulldogs during walk using a pressure sensitive walkway. We hypothesize that Bulldogs affected by orthopedic diseases have decreased weight bearing and asymmetric locomotion in the limbs despite having mild to no sings during clinical examination. Thirty English Bulldogs were tested. Peak vertical force, vertical impulse, rate of loading, stance phase duration, symmetry index, goniometry and incidence of orthopedic diseases were recorded.

**Results:**

Although none of the dogs showed signs of pain or discomfort upon manipulation of the hip joints, all dogs had radiographic evidences of hip dysplasia and lack of significant peak vertical force, vertical impulse and stance time differences. The dogs had a mean hind limb symmetry index of 19.8 ± 19.5% and rates of loading ranged from 1.0 to 3.1.

**Conclusions:**

Despite the lack of evident decrease in weight bearing, subclinical lameness can be inferred. The examined dogs had a mean hind limb symmetry index of 19.8 ± 19.5%. Symmetry indices reported in dogs free from orthopedic diseases range from 0.3 to 9.6%. Given non-lame dogs are expected to have a symmetry index close to 0%, data from this study suggests that Bulldogs have gait dysfunctions, which translates into hind limb asymmetries and rate of loading was consistent with severe hip dysplasia despite no visible signs of gait dysfunction. Future studies comparing lame and non-lame Bulldogs are warranted.

## Background

English Bulldogs are highly predisposed to conformational disorders [1]. This dog breed is actually thought to have the second highest prevalence of orthopedic conditions associated with conformational defects, such as patellar luxation and dysplasia of the elbow and hip joints [2].

Lameness or limited limb function may manifest in a variety of clinical signs, ranging from subtle gait changes to limb function compromise [3]. Kinetic gait analysis permits quantitative assessment of locomotion based on ground reaction forces (GRF) which measures the peak vertical force (PVF) and vertical impulse (VI). These parameters, which have been shown to have 90% sensitivity and specificity for lameness detection, can be accurately measured using pressure sensitive platforms and force plates [4–7]. Other sensitive lameness detection parameters, such as the symmetry index (SI), can also be calculated to help in the diagnosis of canine low grade subtle lameness [8], with the added benefit of eliminating inter-subject variability; since each dog serves as its own control [8].

Orthopedic examination is traditionally based on subjective limb function assessment tools such as numerical and visual analogue scales [9, 10]. However, these methods are thought to be less sensitive compared to kinetic analysis and may fail to accurately characterize lameness [3, 11, 12].

Kinetic analysis studies describing gait parameters in healthy dogs [5, 13, 14], gait characteristics of different dog breeds [13, 15], lameness resulting from different orthopedic conditions [16–20] and force distribution in limb pads of non-lame dogs and dogs suffering from orthopedic conditions [5, 21, 22] have been published.

The establishment of normative gait parameters for healthy canine populations is vital for improved understanding and treatment of orthopedic conditions [23, 24]. Breed-specific locomotion studies are needed to explain the impact of particular conformation features on musculoskeletal function [25]. Factors such as velocity, acceleration, body weight, individual conformation and musculoskeletal structure may influence kinetic variables such as PVF and VI [4, 13, 22, 26]. This study was aimed at assessing kinetic gait parameters in English Bulldogs via pressure sensitive platform analysis. The low accuracy of visual observation in detecting lameness and the lack of studies on chondrodystrophic breeds were the major motivations behind this trial. Our hypothesis was that, given the peculiar conformation of English Bulldogs, gait parameters would differ between this and other dog breeds; wide inter-individual variability was also suspected.

## Methods

### Study population

This project was approved by the CEUA (Ethics Committee for the Use of Animals) of the School of Veterinary Medicine and Animal Science of the University of São Paulo (FMVZ-USP) (CEUA No. 2741210814). Thirty client-owned English Bulldogs were included. The dogs were submitted to a complete physical, orthopedic and radiographic examination prior to kinetic gait analysis. All major muscles, bones and joints of the four limbs were thoroughly examined for crepitus, effusion, pain, discomfort and instability. Informed owner consent was obtained in all cases. Inclusion criteria were as follows: no corticosteroid(s), chondroprotective or non-steroidal anti-inflammatory medication use for a minimum of 4 weeks, absence of systemic diseases, non-pregnant (females), absence of limb tumors or wounds, and absence of neurological disorders.

### Kinetic gait analysis data collection

Dogs were assessed while walking using a pressure distribution recording system which reflects ground reaction forces (Tekscan^®^, 7100 QL Virtual Sensor 3 Mat System, Tekscan Inc. South Boston, MA, USA). This system comprises one platform with three plates arranged in sequence. The platform dimensions are 1.5 × 0.5 × 0.005 m.

Five valid passages out of a maximum of 20 trials were selected per dog. The first four passages were aimed at acclimatization to the equipment and discarded; only complete gait cycles recorded in the middle third of the platform were considered. To limit potential variability, kinetic data collection was always performed in the morning prior to radiographic examination and daily physical activities.

Sensors were calibrated against a known calibration standard weight prior to each test session. All passages started 2 m away from the platform. Standardized velocity ranging from 0.8 to 1.2 m/s was used; velocity control parameters corresponded to stance time (ST) ± 0.1 s between two consecutive footfalls of the same limb and acceleration of ± 0.1 m/s^2^. Dogs were kept on the left side of the handler and led in a straight line with the head looking forward in the direction of travel.

**Fig. 1:**
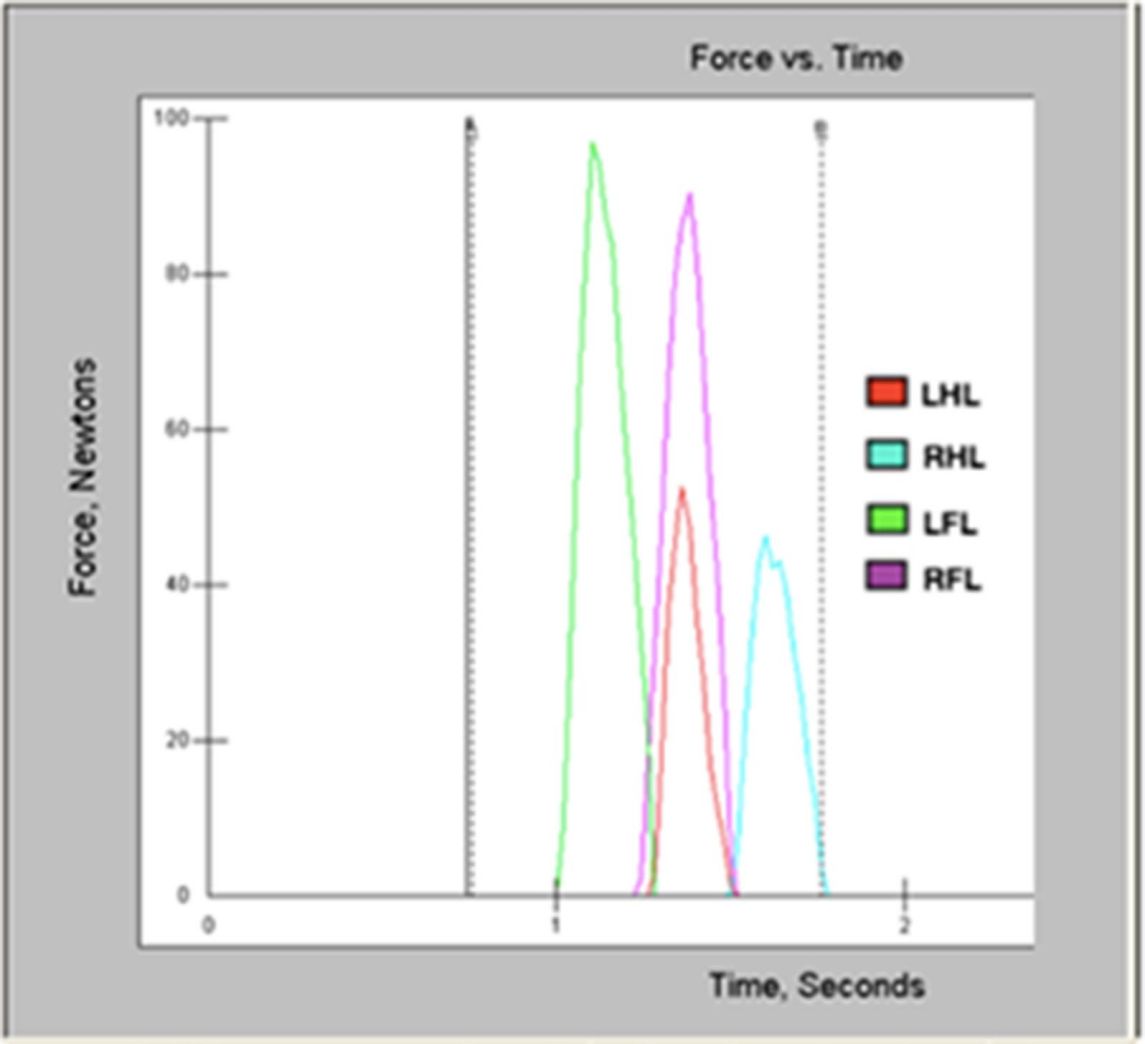
Vertical force graph generated from one valid passage (Dog No. 5) recorded at the Locomotor System Laboratory, FMVZ-USP, São Paulo, 2015. *LHL* left hind limb, *RHL* right hind limb, *LFL* left fore limb, *RFL* right fore limb

Analyses and calculations were based on graphs generated from software (I-scan 5.231, Tekscan Inc., South Boston, MA, USA) (Fig. 1). The following kinetic gait parameters were considered: PVF (Newton, N), VI (Newton seconds, N*s), ST (seconds, s). From the kinetic data, the rate of loading (ROL) and the SI was calculated as follows:

$$\begin{aligned}{\text{ROL}} =&\, {\text{Measured from diagonal pairs of limbs}}, {\text{lowest}}/{\text{highest fore limb PVF}},\\ & \quad {\text{lowest}}/{\text{highest hind limb PVF}}. \end{aligned}$$

$${\text{SI}} = [200*\left( {{\text{highest PVF}} - {\text{lowest PVF}}} \right)/({\text{highest PVF}} + {\text{lowest PVF}})].$$

Other variables included in the analysis were mean body weight, mean age and presence of concurrent orthopedic diseases.

### Statistical analysis

Statistical analyses were based on mean PVF, VI and ST values obtained via baropodometric analysis. The paired *t* test and the Wilcoxon matched-pair test were used in the analysis of data with normal and non-normal distribution respectively. The level of significance was set at 5% (*P* < 0.05).

## Results

Of the 30 English Bulldogs, 18 were males (60%) and 12 were females (40%). Mean body weight and age ± standard deviation (± SD) corresponded to 25.6 ± 4.5 kg and 3.6 ± 2.4 years, respectively. During the physical and orthopedic examinations, none of the dogs showed signs of pain or discomfort upon manipulation of the hip joints. However, according to the radiographic examination, all dogs had radiographic evidences of bilateral hip dysplasia. The radiographic findings were regarded as mild in 66.7% of the dogs, moderate in 16.6%, with 16.6% having severe hip dysplasia.

Mean values did not differ significantly between left and right fore and hind limbs. Mean PVF expressed as percentage of body weight (%BW) and percentage of weight bearing distribution (%WBD), VI (%BW and %WBD) and ST (s and %) values are given in Table 1.

**Table 1:** Gait parameters peak vertical force (PVF), vertical impulse (VI) and stance time (ST) (Mean ± SD) measured in English Bulldogs

Parameter	RFL	LFL	RHL	LHL
PVF (%BW)	37.7 ± 9.1	39.5 ± 9.6	20.5 ± 5.8	21.2 ± 6.5
PVF (%WBD)	29.2 ± 7.8	30.7 ± 7.9	16.0 ± 4.9	16.4 ± 5.2
VI (%BW)	8.4 ± 2.1	9.0 ± 2.4	4.7 ± 1.9	4.7 ± 1.9
VI (%WBD)	31.3 ± 3.0	33.5 ± 3.9	17.5 ± 4.2	17.6 ± 4.3
ST (s)	0.4 ± 0.1	0.4 ± 0.1	0.4 ± 0.1	0.4 ± 0.1
ST (%)	25.6 ± 1.3	25.8 ± 1.2	24.2 ± 1.5	24.3 ± 1.6

*LHL* left hind limb, *RHL*: right hind limb, *LFL* left fore limb, *RFL* right fore limb, *%BW* percentage of body weight, *%WBD* percentage of body weight distribution

Rates of loading measured from FL/HL diagonals ranged from 1.0 to 3.1 (mean 1.9 ± 0.4). Fore and hind limb symmetry indices corresponded to 9.8 ± 7.4 and 19.8 ± 19.5% respectively.

Concurrent orthopedic diseases were detected in 75.0% of the dogs as follows: bilateral patellar luxation (25.0%); elbow dysplasia and unilateral patellar luxation (20.0%); degenerative joint disease (DJD) of the hip and stifle (23%); DJD of the elbow (10.0%); DJD of the carpus and tarsus, elbow trauma and clinical evidences of bilateral or unilateral cranial cruciate ligament rupture (5.0%). Kinetic gait parameters did not differ significantly between dogs presenting exclusively with hip dysplasia and those presenting with additional concurrent orthopedic diseases.

## Discussion

Locomotion system laboratories rely on three interdependent basic elements for gait assessment: human perception, quantitative measurements and biomechanical analysis [27].

Most (83.3%) dog owners in this study failed to report lameness and their dogs did not show signs of discomfort upon examination at the walk or trot. However, kinetic analysis revealed gait changes that were consistent with detected radiographic abnormalities. Clinical lameness diagnosis is subjective in nature. In contrast, objective limb weight bearing quantification via kinetic analysis provides an accurate lameness detection tool. The higher value of force plate analysis compared to numerical rating and visual analogue scales was demonstrated by Quinn [3] in a study involving 21 dogs submitted to right hind limb tibial osteotomy and correction with external fixator. In that study, low levels of inter-rater agreement were reported when numerical rating and visual analogue scales were used.

English Bulldogs are at the top (72.0%) of the Orthopedic Foundation for Animals (OFA) list of dog breeds prone to hip dysplasia [28]. Despite our efforts to get dogs without orthopedic alterations; all dogs in this study were submitted to radiographic screening and had evidences of hip dysplasia with varying degrees of severity. Most of them were also diagnosed with concurrent orthopedic diseases, which may have contributed to increased variability in kinetic data. Despite the implementation of selective breeding programs based on radiography and the reduction in the prevalence of hip dysplasia in some breeds [29, 30], there is poor or no improvement in others [31], suggesting the need for stricter criteria for selection of dogs used for breeding purposes.

The stance phase of the stride is known to be directly related to gait velocity (i.e. the higher the velocity, the shorter the stance phase) [32]. In an effort to reduce velocity-dependent variations, velocity was carefully controlled in this study. Velocity control was also important to prevent passage invalidation since increased walking velocity often led to limb superimposition. This precluded data evaluation.

Lack of significant PVF, VI and ST differences between left and right fore and hind limbs in dogs affected with hip dysplasia in this study may have reflected the bilateral nature of this condition in most cases [33]. Comparative kinetic gait analysis of dogs of different breeds presenting with different degrees of hip dysplasia revealed mean PVF, VI and ST values of 44.0 ± 6.7, 13.1 ± 4.5%BW and 0.48 ± 0.12 s (forelimbs), and 21.7 ± 6.0, 6.3 ± 2.8%BW and 0.45 ± 0.14 s (hind limbs) when dogs were walked at 0.65 ± 0.19 m/s velocity [26]. Lameness severity and duration, as well as the number of joints involved, are known to affect force distribution [4]. Most Bulldogs in this study had concurrent orthopedic diseases which may have accounted for wider kinetic data variability compared to dogs of other breeds affected with hip dysplasia. Ground reaction forces are known to vary widely between lame and non-lame limbs of the same animal [34]. Therefore, comparisons between contralateral limb pairs are not recommended.

The SI is a sensitive and specific parameter for asymmetry assessment in mildly lame dogs. Dogs in this study had mean hind limb SI of 19.8 ± 19.5%. SIs reported in dogs free from orthopedic diseases examined at 0.9 to 1.2 m/s gait velocity range from 0.3 to 9.6% [8]. Hence, subclinical lameness can be inferred despite the lack of evident decrease in weight bearing. Given non-lame dogs are expected to have SIs close to 0%, data from this study suggest Bulldogs have gait dysfunctions which translate into hind limb asymmetries. Such asymmetries and the levels of high individual variability may have accounted for the lack of an inverse relationship between SI and PVF in this trial.

Despite inherent limitations, mean ROL was consistent with severe hip dysplasia despite dogs not presenting with visible signs of gait dysfunction [19]. Consequently, this study underlines the clinical relevance of treating osteoarthritis and respective underlying causes [35] as determined by any possible means.

## Conclusions

The results of this study indicate that English Bulldogs, which present with a high prevalence of orthopedic diseases, also present with a high variability in kinetic data as tested by pressure sensor distribution during a walk. SI was shown to be a potential parameter to assess locomotion in dogs with no evident signs of lameness. Future studies comparing lame and non-lame Bulldogs are warranted.

## Abbreviations

CEUA: Ethics Committee for the Use of Animals
DAD: Degenerative Joint Disease
FMVZ-USP: School of Veterinary Medicine and Animal Science of the University of São Paulo
GRF: ground reaction forces
LFL: left fore limb
LHL: left hind limb
N: Newton
N*s: Newton seconds
OFA: Orthopedic Foundation for Animals
PVF: peak vertical force
RFL: right fore limb
RHL: right hind limb
ROL: rate of loading
SI: symmetry index
VI: vertical impulse
%BW: body weigh
± SD: ± standard deviation
